# FRIENDSHIP MATTERS: UNDERSTANDING THE LINK BETWEEN FRIENDSHIP AND HEALTH IN LATER LIFE

**DOI:** 10.1093/geroni/igad104.1585

**Published:** 2023-12-21

**Authors:** Emily Lim, Katherine Fiori

## Abstract

Friendship matters in later life, as friends provide social support to older adults. Yet, current social relationships and health literature focuses primarily on family relationships. This symposium aims to highlight studies that explore the associations among friendship, health, and health behaviors, with a focus on individual differences. First, using the Health and Retirement Study (HRS), Lim and Burr demonstrate a non-linear relationship between the number of friends and loneliness, with a possible threshold effect for men but a more complex relationship for women. In their investigation of the moderating role of friendship on the association between widowhood and cognition using the HRS, Zhang et al. also find interesting gender differences where greater friendship strain is associated with a faster cognitive decline for widowed men, but not women. Ng and Birditt also uncovers important individual differences, this time in the association between friendship and mental health among Black and White dementia caregivers. They find that emotional support from friends is linked to lower levels of anxiety and depression, but primarily for White caregivers. Mimbs et al. extend these studies to focus on health behaviors, addressing links between interactions with family and friends and hurricane preparedness and evacuation likelihood. Using an online survey of older Floridians, they show that more interactions with friends are associated with greater preparedness and evacuation likelihood. Our discussant, Katherine Fiori, will discuss how these findings can extend the literature on friendship and health in later life, with a focus on the roles of individual differences and context.

